# Xylitol Production: Identification and Comparison of New Producing Yeasts

**DOI:** 10.3390/microorganisms7110484

**Published:** 2019-10-23

**Authors:** Clara Vida G. C. Carneiro, Flávia Cristina de Paula e Silva, João R. M. Almeida

**Affiliations:** 1 Laboratory of Genetics and Biotechnology, Embrapa Agroenergy, Brasilia 70770-901, Brazil; flavia_dipaula@yahoo.br; 2 Graduate Program of Microbial Biology, Department of Cell Biology, Institute of Biology, University of Brasilia, Brasilia 70910-900, Brazil; 3 Graduate Program of Chemical and Biological Technologies, Institute of Chemistry, University of Brasilia, Brasilia 70910-900, Brazil

**Keywords:** xylitol, xylose, hydrolysate, fermentation, sugarcane

## Abstract

Xylitol is a sugar alcohol with five carbons that can be used in the pharmaceutical and food industries. It is industrially produced by chemical route; however, a more economical and environmentally friendly production process is of interest. In this context, this study aimed to select wild yeasts able to produce xylitol and compare their performance in sugarcane bagasse hydrolysate. For this, 960 yeast strains, isolated from soil, wood, and insects have been prospected and selected for the ability to grow on defined medium containing xylose as the sole carbon source. A total of 42 yeasts was selected and their profile of sugar consumption and metabolite production were analyzed in microscale fermentation. The six best xylose-consuming strains were molecularly identified as *Meyerozyma* spp. The fermentative kinetics comparisons on defined medium and on sugarcane bagasse hydrolysate showed physiological differences among these strains. Production yields vary from Y_P/S_ = 0.25 g/g to Y_P/S_ = 0.34 g/g in defined medium and from Y_P/S_ = 0.41 g/g to Y_P/S_ = 0.60 g/g in the hydrolysate. Then, the xylitol production performance of the best xylose-consuming strain obtained in the screening, which was named *M. guilliermondii* B12, was compared with the previously reported xylitol producing yeasts *M. guilliermondii* A3, *Spathaspora* sp. JA1, and *Wickerhamomyces anomalus* 740 in sugarcane bagasse hydrolysate under oxygen-limited conditions. All the yeasts were able to metabolize xylose, but *W. anomalus* 740 showed the highest xylitol production yield, reaching a maximum of 0.83 g xylitol/g of xylose in hydrolysate. The screening strategy allowed identification of a new *M. guilliermondii* strain that efficiently grows in xylose even in hydrolysate with a high content of acetic acid (~6 g/L). In addition, this study reports, for the first time, a high-efficient xylitol producing strain of *W. anomalus*, which achieved, to the best of our knowledge, one of the highest xylitol production yields in hydrolysate reported in the literature.

## 1. Introduction

Xylitol is a five-carbon polyol (sugar alcohol) with different applications. It possesses high sweetener potential, with a flavor similar to sucrose, and it can also be used as a sweetener by patients with diabetes because it does not require insulin to be metabolized [1,2]. Xylitol leads to preventive action against inflammation of the airways, such as otitis, sinusitis and tooth decay since bacteria do not use it for growth [2,3]. It has also been pointed out as an interesting chemical product for worldwide applications in the chemical industry [4].

Currently, xylitol is produced by chemical route, trough the catalytic hydrogenation of xylose, in the presence of an aluminum or nickel catalyst under extreme conditions of temperature and pressure (180 °C; 50 atm) [1,2,3,4,5]. The biotechnological conversion of xylose to xylitol, in yeasts and filamentous fungi, occurs in a single reductive step, where xylose is reduced to xylitol by a xylose reductase (XR) enzyme. The xylitol can be secreted or further metabolized by the microorganism to xylulose by a xylitol dehydrogenase (XDH) enzyme. The biotechnological production of xylitol is attractive in a biorefinery context by the employment of environmental friendly processes to aggregate value to the xylose present in the hemicellulosic hydrolysates [3]. In addition, this process has the potential to offer lower costs for the production routes [6]. 

Yeasts can produce considerable amounts of xylitol, however each species and even different strains of the same species present peculiarities regarding production efficiencies [7,8]. *Candida* species have been recognized as the best xylitol producers, reaching yield and productivity of Y_P/S_ = 0.84 g/g and P = 1.01 g/L h^−^¹, respectively [1]. However, *Candida* species are considered opportunistic pathogens, and their utilization in biotechnological processes have been questioned due to the absence of a GRAS status (generally recognized as safe) [1,2,3]. In this context, several yeast species have been screened and identified, including species of *Meyerozyma* [9], *Spathaspora* [8], *Scheffersomyces* [10], *Debaromyces* [11], and *Kluyveromyces* [12]. Recently, the *Spathaspora* sp. JA1 strain stood out due to its capability to produce xylitol with yields of Y_P/S_ = 0.75 g/g on sugarcane hydrolysate [8]. However, a direct comparison among the xylitol producers is difficult because of the different experimental setups employed in each study. 

Despite the availability of xylitol-producing strains, the identification of yeasts that are naturally able to produce xylitol and the systematic comparison among producing strains, is still necessary, so that the demand for specific substrates and large-scale production conditions are supplied [5,6,7,8,9,10,11,12,13]. In this context, the aim of this study was to select and identify a new yeast naturally able to convert xylose from sugarcane bagasse hydrolysate into xylitol and then to compare its performance with strains previously known as good xylitol producers.

## 2. Materials and Methods 

### 2.1. Yeast Strains 

The 960 screened yeasts used in this work were isolated from soil, decaying wood, and insect guts, collected at Brasília, Federal District, Brazil, and were deposited in the microbial collection of Embrapa Agroenergia. The yeasts were stored in a −80 °C freezer in Yeast Mold (YM) medium supplemented with xylan. The yeasts were stored in microtiter plates, which were named from JAP5 to JAP14. The yeasts were isolated from different environments: JAP5 and JAP14 from decaying wood, JAP6 and JAP7 from termites, JAP8 and JAP9 from insect larvae growing in decaying wood, JAP10, JAP11, and JAP12 from deeper soil (around 5 cm from the surface), and JAP13 from superficial soil. Three yeast species previously identified were also chosen as control strains for fermentative kinect comparison: (i) *Spathaspora* sp. JA1 was previously isolated from decaying wood samples collected in Brasilia, Distrito Federal, Brazil, by its capacity to grow on xylose. The capability of *Spathaspora* sp. JA1 to produce xylitol on hydrolysate was recently demonstrated [7]; *Meyerozyma guilliermondii* A3 was isolated by its fast growth on xylose and capability to produce xylitol on sugarcane bagasse hydrolysate [14]; and, finally, *Wickerhamomyces anomalus* 740 isolated from a sugarcane mill sample by its ability to grow on sugarcane bagasse hydrolysate containing high concentration of acetic acid (8 g/L). This strain was capable to metabolize glucose and xylose and produce mainly cell biomass, ethanol, and xylitol in a defined mineral medium and sugarcane bagasse hydrolysate. 

### 2.2. Media

The media used were: Yeast Mold (YM) medium (yeast extract 3 g/L, glucose 10 g/L, bacterial peptone 5 g/L, and malt extract 3 g/L); YPD (yeast extract 10 g/L, bacterial peptone 20 g/L, and glucose 20 g/L) and YPX (yeast extract 10 g/L, bacterial peptone 20 g/L, and xylose 20 g/L). The defined medium was composed of YNB (synthetic YNB 1.7 g/L Sigma Aldrich Y1251 and ammonium sulfate 5.0 g/L) supplemented with xylose 40 g/L or glucose 20 g/L. Mineral medium (MM) was prepared as reported by [15] and it was supplemented with sugarcane bagasse hydrolysate, or xylose (40 g/L) and glucose (10 g/L). 

### 2.3. Screening of Naturally Xylose-Consuming Yeasts

The yeasts were screened for the ability to grow in defined medium containing xylose as the sole carbon source. For this, the strains were withdrawn from −80 °C stock and inoculated in 200 µL of YM medium supplemented with glucose 20 g/L in 96 well microtiter plates (numbered as JAP5 to 14) and incubated at 30 °C overnight. After confirmation that all strains grew on glucose, they were replicated in 200 µL of defined medium supplemented with xylose (40 g/L). The transfer was made using a sterile 96 pin Replicator (Boekel, Pennsylvania, PA, USA). The plates were incubated at 30 °C and the growth was monitored by optical density measures (OD_600_ nm) using a spectrophotometer (Molecular Devices SpectraMax M3). The collected data were evaluated to quantify the capacity for growth of each strain. The experiments were made in quadruplicate. The yeasts *Scheffersomyces stipitis* NRRL and *Spathaspora* JA1 were used as positive controls because of their ability to grow on xylose [8,9,10,11,12,13,14,15] in all experiments, whereas media without inoculum was used as the negative control. To visually represent the final growth of each strain, a heat map was constructed. For this, the calculated growth of each strain was calculated by absorbance variation in 120 h (OD_600_ 120 h–OD_600_ 0 h). The best growing strain showed a growth at 120 h of OD_600_ of 1.47. This value was considered 100% and used to normalize the growth of all strains. To perform the selection of the best strains, the following criteria were used: (i) identification of strains that reach higher OD_600_ nm, regardless of time, and (ii) identification of strains with faster growth; that is, reach the stationary phase more quickly. In addition, to ensure diversity of strains origin, yeasts of all plates, except one, have been selected for the next experiments. 

### 2.4. Fermentative Analysis in Microscale

The xylose conversion capability of 42 selected strains was evaluated in 200 µL of YM supplemented with xylose, inoculated with low cell density, in a 96 wells microtiter at 30 °C, overnight. After yeast growth in the preculture, they were replicated to deepwell plates (1.1 mL) containing 800 µL of defined medium supplemented with xylose 40 g/L using a 96 pins sterile replicator (Boekel, USA) and incubated at 30 °C for 48 h. After that period, the plates were centrifuged for 20 min at 3800× *g* and the supernatant was collected for analysis of sugar consumption and product formation. 

After the previous experiment, the eight best xylose-consumers and xylitol producers were selected for fermentation with higher cellular density. For this, the yeast strains were inoculated in 10 mL of a defined medium supplemented with xylose 40 g/L in conical tubes of 50 mL and incubated at 180 rpm at 28 °C overnight. Then, the tubes were centrifuged and the cells were washed and inoculated in 1 mL of a defined medium in deepwell plates (1.1 mL). After incubation for 48 h at 28 °C, the supernatants were collected for analysis of sugar consumption and product formation.

### 2.5. Fermentative Capacity Evaluation Under Limited-Oxygen Conditions 

#### 2.5.1. Comparison of Xylitol Production by Selected Yeasts in a Defined Medium

A total of six yeast strains was chosen to perform high cell density fermentation in flasks. The six *Meyerozyma* sp. strains were grown in 50 mL conic tubes, with 7 mL of a defined medium supplemented with xylose (40 g/L), at 180 rpm and 28 °C, overnight. Then, the strains were inoculated to an OD_600_ nm of 0.2 in 50 mL of a defined medium in 100 mL flasks, and incubated at 180 rpm and 28 °C, for 72 h. Samples were withdrawn for sugar consumption and product formation measurements. The experiment was made in duplicate.

#### 2.5.2. Comparison of Xylitol Production by Selected Yeasts in Sugarcane Bagasse Hydrolysate

The yeasts *M. guilliermondii* B12, *M. guilliermondii* A3, *Spathaspora* sp. JA1, and *Wickerhamomyces anomalus* 740 were grown overnight in 150 mL of YPX in 1 L flasks at 180 rpm, 28 °C. Then, 500 mL of sugarcane hydrolysate supplemented with xylose (12 g/L to reach 40 g/L) and glucose (2 g/LL to reach 10 g/L) in a 1 L bench bioreactor (Infors MT) was inoculated to an OD_600_ nm of 1. The hydrolysate was prepared by steam explosion followed by acid hydrolysis of the hemicellulosic fraction of sugarcane bagasse, and the composition in the beginning of fermentation was determined to be: cellobiose (0.56 g/L), glucose (10 g/L), xylose (40 g/L), acetate (6.5 g/L), furfural (0.94 g/L), and 5-hydroxymethylfurfural (5-HMF) (0.11 g/L). 

The fermentation was carried out at 28 °C, stirrer at 400 rpm, and a pH of 5.5 adjusted with KOH 3 M. Synthetic air was flushed during the experiment at 0.2 v.min^−^¹. The experiments were made in triplicate.

### 2.6. Yeast Molecular Identification 

Genomic DNA was extracted using a yeast genomic DNA extraction kit (Invitrogen). The region D1/D2 of 26S ribosomal DNA was amplified by PCR using the primers NL1 and NL4 [16]. The PCR products were purified using a DNA purification kit (Thermo Scientific, Waltham, MA, USA) and sanger sequenced by Eurofins Scientific. The consensus sequence for each strain was constructed using the Geneious program. Finally, the sequence of each yeast (Table S1) was compared with the sequences of the D1/D2 region of the strains belonging to NCBI databases and CBS–KNAW Collections for taxonomic identification.

### 2.7. Metabolite and Biomass Analyses

Metabolite samples were immediately centrifuged and stored at −20 °C until analysis by liquid chromatography. An HPLC-UPLC (Waters AcQuity UPLC H Class) equipped with an Aminex HPX 87H column (Bio-Rad, Hercules, CA, USA) at 45 °C and a refractive index detector (Waters 410, Millipore, Milford, MA, USA) was employed to quantify glucose, xylose, xylitol, glycerol, acetate, and ethanol. The elution was performed with 5 mM H_2_SO_4_ prepared in ultra-pure water with a flow rate of 0.6 mL/min.

Cell concentrations were determined from absorbance measurements at 600 nm on samples diluted to give an optical density (OD) using a spectrophotometer (Molecular Devices SpectraMax M3). For the determination of cell dry weight, duplicate 5 mL samples, which were centrifuged, washed with distilled water, and dried at 60 °C for 48 h, were weighed. Cell dry weight was linearly correlated with OD measurements. 

## 3. Results

### 3.1. Bioprospecting Xylitol Producing Yeasts

**Figure 1:**
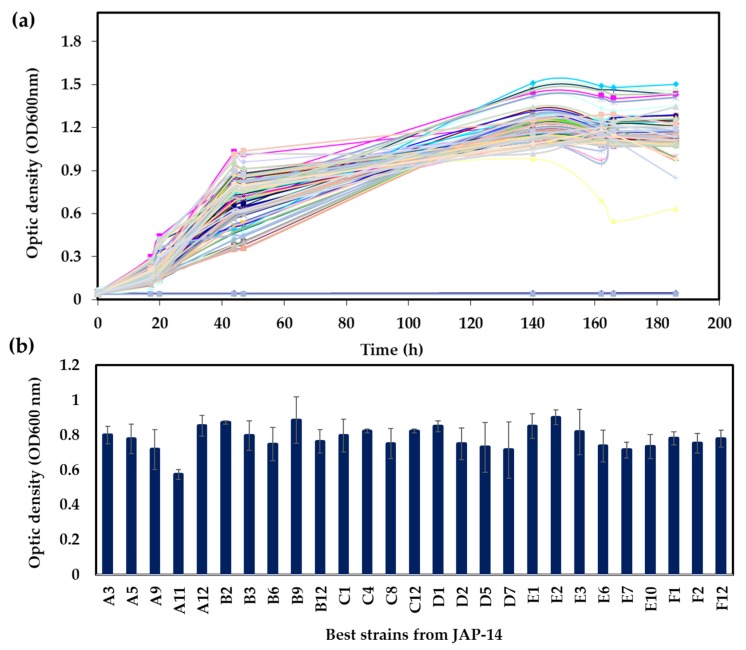
Selection of yeasts based on growth in xylose. (**a**) Growth profile of the 96 yeasts in defined medium. The colored lines represent the different strains of the plate JAP-14 (**b**) Final growth in 180 h for the 30 best strains in the microtiter plate JAP-14. Average and standard deviation for four replicates are shown.

**Figure 2:**
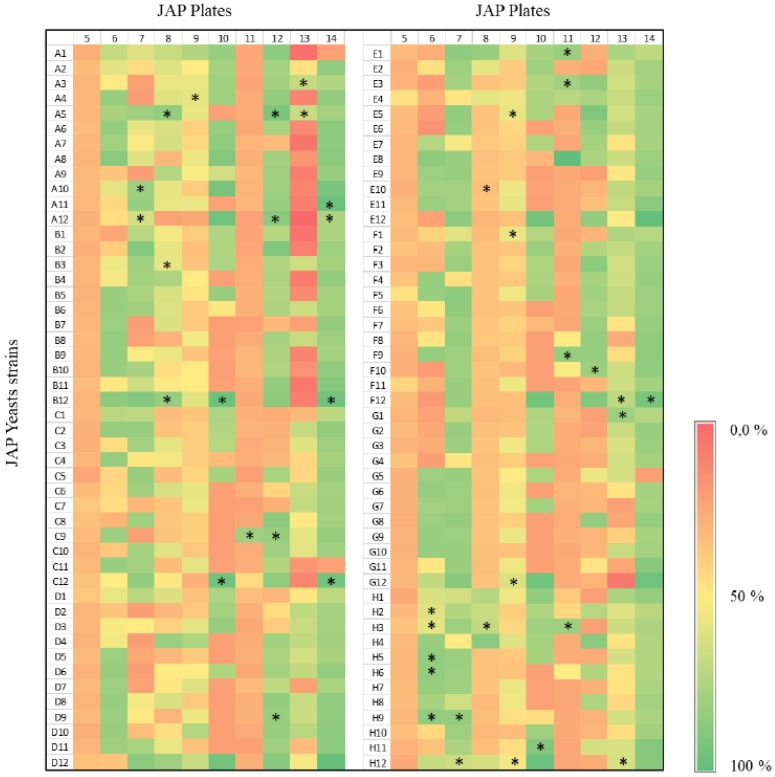
Heat Map showing the growth score of the 960 yeast strains screened on defined medium supplemented with xylose after 120 h. The lines represent the yeast strains code in each plate, whereas the columns the plate numbers. All the values were normalized with the highest growth (OD_600_ 120 h–OD_600_ 0 h); i.e., 100% equals to OD_600_ 1.47. The 42 selected yeast strains are indicated by *.

A screening strategy based on the ability to metabolize xylose was employed to select yeast strains to produce xylitol. Initially, 960 yeasts isolated in the Cerrado biome in Brazil were evaluated by growth in xylose as the sole carbon source in microtiter plates. The growth profile varied considerably among the different yeasts (Figure 1 and Figure 2). While some strains have reached the stationary phase after 24 h with relatively low cell density, others did grow slowly but reached higher cell densities. Figure 1 exemplifies the growth profile for 96 strains. In addition, the final OD_600_ reached after 24 h was scored for all strains (Figure 1b). Strains isolated from different environments showed significant growth capacities on xylose (Figure 2; Table S2). In general, strains isolated from JAP5 and JAP11 showed the poorest growth on xylose, while JAP10, JAP12, and JAP14 showed the best growth capacity (Figure 2). As the preculture was performed in glucose and the growth of each strain was confirmed, these results are in fact related to the ability to metabolize xylose under the conditions evaluated. 

Two criteria were employed to select the best growing yeasts from each plate: (i) selection of strains that reach a higher final growth, regardless of time, and (ii) a selection of strains with faster growth; that is, reach the stationary phase in a shorter time. To ensure diversity of strain origin, yeasts of all plates have been selected for the next experiments, with the exception of JAP5, none of the yeasts of the plate JAP5 presented a growth rate above 50% in comparison with the best strain. Therefore, they were excluded from the next experiments. Thus, after the growth analysis of all yeasts, the 42 best-growing strains were selected. Among these, 9 were isolated from termite samples, 10 came from termite larvae, 13 from deep soil, 5 from superficial soil, and 5 from decaying wood (Figure 2).

**Figure 3:**
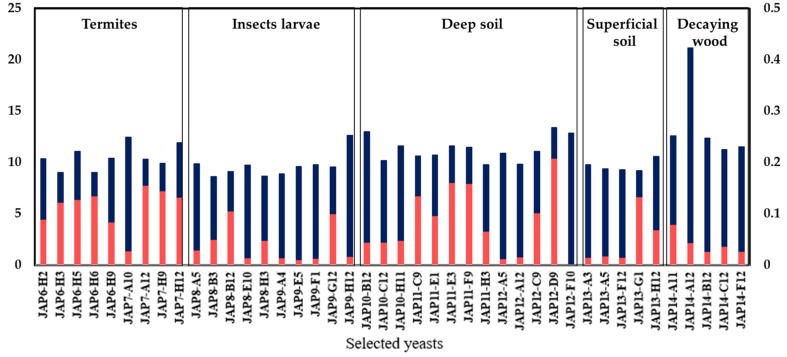
Xylose consumption and xylitol production after 48 h by the 42 selected yeast strains inoculated at a low cellular density in a defined medium supplemented with 40 g/L xylose. Grey lines (consumed xylose); black lines (xylitol production). The eight selected yeast strains are indicated by *.

The sugar consumption and xylitol production of the 42 selected strains were evaluated in a defined medium supplemented with xylose (40 g/L). As expected, all strains were able to consume xylose; however, with significant variation among them (Figure 3). The strain JAP14-A12, which was isolated from a decaying wood sample, consumed the highest amount of xylose (20 g/L), whereas the JAP8-B3 strain showed the poorest consumption (8.5 g/L). The origin of the yeast did not appear to correlate with xylose consumption capacity, since significant variation in the xylose consumption occurred even among the yeasts isolated from the same origin samples. 

A total of 41 strains were able to produce detectable amounts of xylitol among the 42 evaluated (Figure 3). The xylitol production was relatively low under the conditions evaluated (low cellular density), with the strain JAP12-D9 producing the highest amount (0.2 g/L). The strains JAP7-H12, JAP6-H5, and JAP8-B12 also showed good xylitol production (0.13 g/L, 0.12 g/L, and 0.10 g/L, respectively) even if they did not show the highest xylose consumption (Figure 3), which may lead to good production yields. Despite the yeast JAP14-A12 showing the highest xylose consumption, it showed a low xylitol production (0.04 g/L). Even if the aeration was limited, the conditions employed still favored yeast growth (data not shown). Based on the presented data, the eight yeast strains that most consumed xylose and produced greater quantities of xylitol were selected for further characterization. Four yeast (JAP6-H2, JAP6-H5, JAP6-H9, and JAP7-H12) were derived from termites, two (JAP8-B12, JAP9-G12) from insect larvae, one (JAP12-D9) from deep soil, and one (JAP14-A12) from decaying wood. 

**Figure 4:**
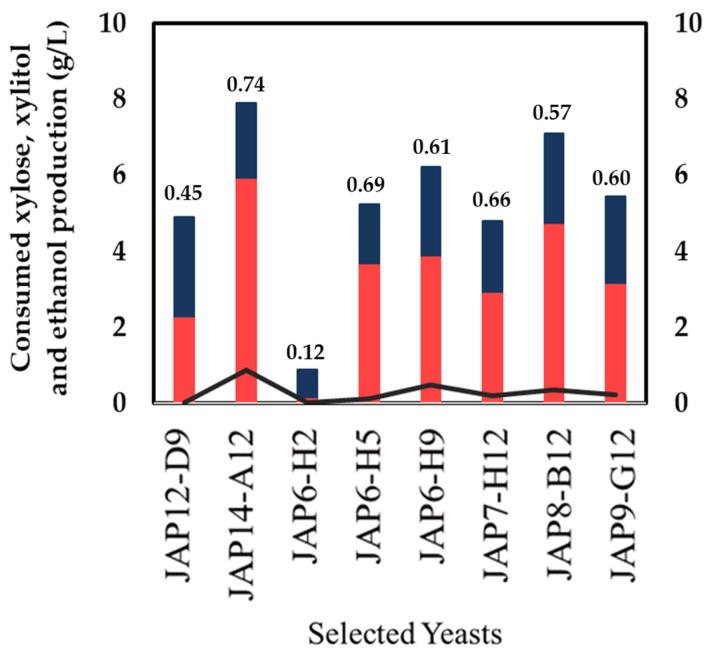
Xylose consumption and xylitol production in a defined medium supplemented with xylose (40 g/L), inoculated at a high cellular density condition, by the eight yeasts previously selected. The yield (g xylitol/g xylose consumed) is represented by the numbers on the top of the columns. Xylose consumption (grey bars); xylitol production (black bars); ethanol production (light grey line).

The eight yeast strains were evaluated for xylitol production with higher cellular density under oxygen-limited condition. The yeast strains were incubated in a defined medium supplemented with xylose 40g/L, and after 72 h the sugar consumption and production formation were analyzed. The JAP14-A12 strain that was isolated from insect larvae showed the best consumption of xylose (8 g/L) and xylitol production (6 g/L), with a yield of Y_X/S_ = 0.74 g/g on xylitol production, followed by JAP7-H12 and JAP6-H9, producing 4.70 g/L and 3.81 g/L with yield of Y_X/S_ = 0.66 g/g and Y_X/S_ = 0.61 g/g, respectively (Figure 4). Five yeast strains were isolated from insects, JAP6-H5, JAP6-H9 and JAP7-H12 isolated from termite viscera, and JAP8-B12 and JAP9-G12 of insect larvae, and then one strain, *M. guilliermondii* A12, from decaying wood. All six strains produced trace amounts of ethanol under the tested conditions. Only two yeasts, JAP12-D9 and JAP6-H2, isolated from the soil and decaying wood samples, respectively, showed the poorest growth, and a yield on xylitol production lower than Y_X/S_ = 0.50 g/g. Therefore, they were excluded from the following experiments.

### 3.2. Taxonomic and Molecular Identification

The six selected yeast strains were molecularly identified by sequencing and analysis of the region D1/D2 of the ribosomal DNA 26S. The analysis of similarity employing data deposited in the NCBI and CBS–KNAW Collections databases described showed that all the six selected strains belong to the genus *Meyerozyma* (Table 1). Five strains showed complete sequence identity to *M. guilliermondii* strains, whereas one showed a 99% identity (Table 1). The strain *M. guilliermondii* G12 showed sequence identity to *M. guilliermondii* and *M. caribbica*, both with 99% identity (Table 1). After the taxonomic identification, the selected yeasts were called by its scientific nomination, plus the final code, i.e., *M. guilliermondii* B12.

### 3.3. Xylitol Production in Defined Medium and Sugarcane Bagasse Hydrolysate

The xylitol production kinetics of the six yeast strains previously selected (*M. guilliermondii* A12, *M. guilliermondii* B12, *M. guilliermondii* G12, *M. guilliermondii* H5, *M. guilliermondii* H9, and *M. guilliermondii* H12) was further evaluated in shake-flasks under oxygen-limited conditions in a defined medium. All six strains presented similar fermentation kinetics, consuming about 25 g/L of xylose in 72 h and producing mainly cell biomass and xylitol (Table 2). The strains *M. guilliermondii* H9 and *M. guilliermondii* B12 were the best xylitol producers, with a production of approximately 7 g/L and yield of approximately 0.32 g/g. Thus, *Meyerozyma guilliermondii* B12, isolated from insect larvae, was selected as the best producer of xylitol among the 960 prospected, to carry the fermentation on sugarcane bagasse hydrolysate.

### 3.4. Xylitol Production in Sugarcane Bagasse Hydrolysate

**Figure 5:**
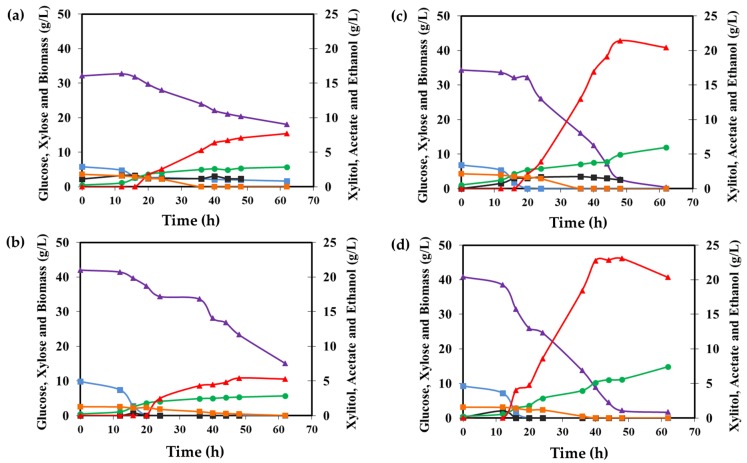
Xylitol production in sugarcane bagasse hydrolysate in a bench bioreactor. (**a**) *M. guilliermondii* A3, (**b**) *M. guilliermondii* B12, (**c**) *Spathaspora* sp. JA1, and (**d**) *W. anomalus* 740. Xylose (purple line), xylitol (red line), glucose (orange line), ethanol (black line), acetate (blue line), and biomass (green line). Experiments were performed in duplicate and one replicate is shown.

The xylitol production capability of the new *M. guilliermondii* B12 strain selected in this study was compared to the one of *M. guilliermondii* A3, *Spathaspora* sp. JA1 and *W. anomalus* 740 in sugarcane bagasse hydrolysate. *M. guilliermondii* A3 was also isolated from the Cerrado biome in Brazil, whereas *W. anomalus* 740 was isolated from a sugarcane mill and presents a high tolerance to acetic acid. *Spathaspora* sp. JA1 was recently identified as a promising xylitol producing yeast [8]. The four strains were inoculated in hydrolysate and the sugar consumption and product formation were evaluated under oxygen-limited conditions. All four yeasts consumed the glucose (aproximatelly 8 g/L) in the first 15 hours of fermentation (Figure 5).

Xylose assimilation only started after the glucose was almost completely consumed (Figure 5). The strains *M. guilliermondii* B12 and *M. guilliermondii* A3 showed a slower sugar consumption rate than *Spathaspora* sp. JA1 and *W. anomalus* 740. Indeed, they spent more time adapting in the hydrolysate medium and only consumed approximatelly 20 g/L xylose after 62 h of the fermentation process and produced approximatelly 5–6 g/L of xylitol (Figure 5a,b and Table 3). On the other hand, *Spathaspora* sp. JA1 and *W. anomalus* 740 showed similar fermentative profile, consuming all the xylose in 50 h (Figure 5c,d) and producing maximum xylitol production at 44 h with 21 g/L and 24 g/L, respectively. These resulted in xylitol producing yields above 0.80 g/g (Table 3). After 50 h, there was no more carbon source in the culture media. However, the concentration of xylitol started to decrease, suggesting that the yeasts began to assimilate the xylitol in the absence of other carbon sources available (Figure 5c,d). All the four yeasts did grow well under the conditions evaluated. The yeast *Spathaspora* sp. JA1 and *W. anomalus* 740 consumed more sugar and produced more cell biomass (Figure 5 and Table 3). 

## 4. Discussion

Lignocellulosic bagasse-derived sugars can be employed for a variety of biotechnological processes. The sugarcane bagasse is one of the most abundant and available residues derived from the bioethanol production in Brazil [17,18,19]. One of the biggest challenges regarding its use is the conversion of xylose in the presence of inhibitory compounds present in the lignocellulosic biomass hydrolysate, such as acetic acid, one of the major inhibitory compounds present in the sugarcane hydrolysate [20]. As the most utilized yeast in ethanol production, *S. cerevisiae* cannot ferment pentoses, and natural xylose-consuming yeasts have been extensively evaluated for xylitol production, including *Meyerozyme* sp. [21], *Debaromyces* [22], *Hansenula* sp. (*Wickehamomyces)* [23], *Kluyveromyces* [24], as well as others such as *Scheffersomyces* [10]. Thus, in this work, we screened for new yeast strains capable of producing xylitol and compare their performance in different experimental setups. 

The bioprospecting of 960 isolated yeasts from soil, decaying wood, and insects resulted in the selection of six *Meyerozyma* strains that are able to consume xylose and produce xylitol. Five strains were identified as *M. guilliermondii* and one could not be differentiated between *M. guilliermondii* and *M. caribbica*. It was shown that these two strains have an almost identical fermentation profile and are identified by its characteristic of producing coenzyme-Q9, and the separation between them is uncertain [25,26]. The species of the genus *Meyerozyma* belong to the phylum Ascomycota and are inserted in the clade Saccharomycotina CTG. However, the phylogenetic structure of the yeasts of this group is not fully understood [24,25,26,27]. Even if the scientific advances enabled the proper identification of some yeasts from this clade, it was pointed out that one of the major problems in this identification is the range of incorrect or non-updated taxonomic annotations in public databases addressing *Meyerozyma* species [28,29].

Although, the screened microbial collection has a variety of yeast genera, such as *Candida*, *Spathaspora*, *Pichia*, and *Wickerhamomyces* [7], and all of the selected yeasts in this work were identified as the genus *Meyerozyma*. This fact can be explained by the ubiquity of the species of this genus, they can be isolated from different environments [30] (thermal waters, fruits, insects, and soil), as well as standing out for its ability to grow fast on pentoses, such as xylose [24,25,26]. Indeed, a previous screening based on the capability to tolerate sugarcane bagasse hydrolysate with a high content of acetic acid (~7 g/L), employing some of the same strains of this study, resulted in the selection of *Candida tropicalis* strains [7]. With this in mind, it is possible to relate that among the 960 yeasts in the collection, the specific *Meyerozyma* strains were selected by the capability to grow fast in culture medium containing xylose as the only carbon source.

The strains *M. guilliermondii* B12 and *M. guilliermondii* A3 employed in this study produced xylitol in relatively high yields considering that the production process was not optimized for sugarcane biomass hydrolysate (Table 3). Under similar fermentation conditions, in a previous work, the yeast *C. guilliermondii* FTI 20037 produced xylitol with a yield of 0.50 g/g in hydrolysate supplemented with glucose (10 g/L) and urea (4 g/L), which is similar to the one registered for *M. guilliermondii* A3 and B12 (Y_X/S_ = 0.60 g/g and 0.42 g/g, respectively) (Table 3) [31]. Another study has reported even lower production yields for xylitol production in non-detoxified (Y_X/S_ = 0.19 g/g) and detoxified hydrolysate (Y_X/S_ = 0.14 g/g) *Meyerozyma* strains [21]. Despite the relatively high xylitol production yields for *M. guilliermondii* B12 and A3, these strains could not completely overcome the hydrolysate toxicity and showed reduced xylose consumption (Figure 5 and Table 3). This may be associated with the acetic acid present in the sugarcane bagasse hydrolysate (~6 g/L), which has been shown to inhibit xylose metabolism in *C. guilliermondii* (*M. guilliermondii*), even in low concentrations (~4 g/L), as well as in other yeasts [32].

Contrary to *M. guilliermondii* strains, *Spathaspora* sp. JA1 and *W. anomalus* 740 were not strongly inhibited by the toxicity of the hydrolysate. Indeed, the yeasts *W. anomalus* 740 and *Spathaspora* sp. JA1 were able to consume the complete xylose present in the culture and showed xylitol production yields above 0.75 g/g (Figure 5c,d and Table 3). The yeast *W. anomalus* is extensively studied for its antimicrobial [33,34], thermotolerance [35], and biosurfactant production [36]. To the best of our knowledge, this work reports the best xylitol production yield by a strain of *W. anomalus* in the literature. Previously, Zha [37] has selected yeast strains for their tolerance to hydrolysate inhibitors, and found a strain of *Pichia anomala* 29X (*W. anomalus*) able to grow using xylose in sugarcane bagasse, but the yeast was not able to produce xylitol. 

The strain *W. anomalus* 740 was able to produce 24.75 g/L of xylitol with a yield of Y_X/S_ = 0.83 g/g (Table 3) in sugarcane bagasse hydrolysate with approximately 6 g/L of acetic acid, inoculated with a low cell concentration. These values stand out in the literature and are comparable with the best xylitol production yields in hydrolysate reported in the literature [3,4,5,6,7,8]. *C. guilliermondii* FT120037 in the presence of high xylose concentration (~90 g/L) in rice straw hydrolysate, achieved a yield of Y_X/S_ = 0.84 g/g [38]. Morais [7] reported a yield of Y_X/S_ = 0.86 g/g for xylitol production by *C. tropicalis* JA2 in sugarcane bagasse hydrolysate with ~7 g/L of acetic acid in a fermentation with a high cellular concentration. Prakash [39] also published a high yield for xylitol production (Y_X/S_ = 0.82 g/g) with immobilized cells of the yeast *Debaryomyces hansenii* in the presence of a high (100 g/L) xylose concentration in sugarcane bagasse hydrolysate.

The yeast *Spathaspora* sp. JA1 showed xylitol production yields similar to previously published work (Y_X/S_ = 0.74 g/g) [8], confirming its potential to be a xylitol producer. Other *Spathaspora* spp. strains have been reported to be able to produce xylitol with good yields [13]. However, the strain *Spathaspora* sp. UFMG-XMD-16.2 reached a xylitol production yield of 0.57 g/g [13], while the strains *S. roraimanensis* UFMG-CM-Y477 and *S. brasiliensis* UFMG-CM-Y353 reached 0.56 g/g and 0.47 g/g, respectively [40].

## 5. Conclusions

A screening strategy based on yeast capability to grow on xylose as the sole carbon source was employed to prospect a collection of 960 strains. The six best xylose-utilizing strains obtained in the screening were identified as *Meyerozyma* spp. Physiological comparisons demonstrated differences among the six strains and pointed out the potential of *M. guilliermondii* B12 for xylitol production on a defined medium and sugarcane bagasse hydrolysate. In addition, systematic comparison of yeast performances in hydrolysate confirmed the potential of *Spathaspora* sp. JA1 for xylitol production and demonstrates, for the first time, the potential of *W. anomalus* as an excellent xylitol producer with yield as high as 0.83 g/g on sugarcane hydrolysate.

## Figures and Tables

**Table 1:** Taxonomic identification of the selected yeast strains. The D1/D2 region sequence similarity is indicated.

Yeast Code	Yeast	Sequence ID	ID
JAP14-A12	*Meyerozyma guilliermondii* MSRY_19	KY952849.1	100%
JAP8-B12	*Meyerozyma guilliermondii* MSRY_19	KY952849.1	100%
JAP9-G12	*Meyerozyma guilliermondii* N2-1 *Meyerozyma caribbica*	MF148904.1 KX507035.1	99% 99%
JAP6-H5	*Meyerozyma guilliermondii* DGC-G-z	MG518185.1	100%
JAP6-H9	*Meyerozyma guilliermondii* 2A-1C315III	MG736036.1	100%
JAP7H12	*Meyerozyma guilliermondii* MSRY_19	KY952849.1	100%

**Table 2:** Xylitol production by selected *Meyerozyma* strains in shake-flasks under oxygen-limited conditions in a defined medium. Xylitol production, yield, and productivity after 72 h of incubation. Values are average and deviation from duplicate experiments.

Microorganism	Xylose Consumed (g/L)	Xylitol Production (g/L)	Xylitol Yield (g/g) ^a^	Productivity (g//g h^–^¹) ^b^
*M. guilliermondii* H5	24.28 ± 5.65	6.69 ± 1.44	0.28 ± 0.01	0.009 ± 0.004
*M. guilliermondii* H9	20.64 ± 2.12	7.19 ± 1.38	0.35 ± 0.03	0.009 ± 0.003
*M. guilliermondii* H12	24.50 ± 2.46	6.20 ± 0.63	0.28 ± 0.07	0.009 ± 0.001
*M. guilliermondii* A12	24.67 ± 4.39	6.37 ± 2.27	0.25 ± 0.05	0.010 ± 0.005
*M. guilliermondii* B12	24.10 ± 0.70	7.25 ± 0.46	0.30 ± 0.02	0.011 ± 0.002
*M. guilliermondii* G12	21.77 ± 2.87	6.19 ± 0.43	0.29 ± 0.02	0.009 ± 0.003

^a^ Y_X/S_: xylitol yield on xylose; ^b^ Volumetric productivity.

**Table 3:** Fermentative parameters for the xylitol-producing yeasts *M. guilliermondii* A3, *M. guilliermondii* B12, *Spathaspora* sp. JA1, and *W. anomalus* 740 in sugarcane bagasse hydrolysate. Values represent average and standard deviation of duplicates values calculated at 44 h of the experiment.

Microorganisms	Xylose Consumed (g/L)	Xylitol Production (g/L)	Xylitol Yield (g/g) ^a^	Productivity (g/g·h^−1^) ^b^	Ethanol Production (g/L)	Ethanol Yield (g/g) ^a^	Biomass Yield (g/g) ^c^
*M. guilliermondii* A3	11.68 ± 0.10	6.12 ± 0.59	0.60 ± 0.01	0.155 ± 0.017	0.83 ± 0.03	0.08 ± 0.00	0.29 ± 0.02
*M. guilliermondii* B12	15.00 ± 3.14	5.00 ± 0.42	0.42 ± 0.08	0.014 ± 0.001	0.87 ± 0.09	0.14 ± 0.06	0.39 ± 0.03
*Spathaspora* sp. JA1	31.29 ± 4.15	21.09 ± 1.97	0.74 ± 0.03	0.057 ± 0.006	3.39 ± 0.11	0.18 ± 0.07	0.24 ± 0.01
*W. anomalus* 740	33.07 ± 3.21	24.75 ± 1.70	0.83 ± 0.13	0.101 ± 0.035	n.d.	n.d.	0.23 ± 0.01

^a^ Y_X/S_, Y_E/S_: xylitol and ethanol yield on xylose, respectively; ^b^ Volumetric productivity; ^c^ Y_B/S_: biomass yield on xylose and glucose. n.d. not detected.

## Supplementary Materials

Supplementary materials can be found at https://www.mdpi.com/2076-2607/7/11/484/s1.
