# Supplementary material for: Xylitol Production: Identification and Comparison of New Producing Yeasts
Source: Microorganisms. 2019 Oct 23;7(11):484. doi: 10.3390/microorganisms7110484 (PMC6920771; doi:10.3390/microorganisms7110484)
Supplement: Supplementary file 1 [file microorganisms-07-00484-s001.zip › supplementary/Table s1- Fasta sequences.docx]

Supplementary material:

A12 - *Meyerozyma guilliermondii* MSRY_19

ID: KY952849.1

100% of identity

F

TAGTAGCGGCGAGTGAAGCGGCAAAAGCTCAAATTTGAAATCTGGCGCCTTCGGTGTCCGAGTTGTAATTTGAAGATTGTAACCTTGGGGTTGGCTCTTGTCTATGTTTCTTGGAACAGGACGTCACAGAGGGTGAGAATCCCGTGCGATGAGATGCCCAATTCTATGTAAGGTGCTTTCGAAGAGTCGAGTTGTTTGGGAATGCAGCTCTAAGTGGGTGGTAAATTCCATCTAAAGCTAAATATTGGCGAGAGACCGATAGCGAACAAGTACAGTGATGGAAAGATGAAAAGAACTTTGAAAAGAGAGTGAAAAAGTACGTGAAATTGTTGAAAGGGAAGGGTTTGAGATCAGACTCGATATTTTGTGAGCCTTGCCTTCGTGGCGGGGTGACCCGCAGCTTATCGGGCCAGCATCGGTTTGGGCGGTAGGATAATGGCGTAGGAATGTGACTTTACTTCGGTGAAGTGTTATAGCCTGCGTTGATGCTGCCTGCCTAGACCGAGGACTGCGATTTTATCAAGGATGCTGGCATAATGATCCCAAACCG

R

CAGTCCTCGGTCTAGGCAGGCAGCATCAACGCAGGCTATAACACTTCACCGAAGTAAAGTCACATTCCTACGCCATTATCCTACCGCCCAAACCGATGCTGGCCCGATAAGCTGCGGGTCACCCCGCCACGAAGGCAAGGCTCACAAAATATCGAGTCTGATCTCAAACCCTTCCCTTTCAACAATTTCACGTACTTTTTCACTCTCTTTTCAAAGTTCTTTTCATCTTTCCATCACTGTACTTGTTCGCTATCGGTCTCTCGCCAATATTTAGCTTTAGATGGAATTTACCACCCACTTAGAGCTGCATTCCCAAACAACTCGACTCTTCGAAAGCACCTTACATAGAATTGGGCATCTCATCGCACGGGATTCTCACCCTCTGTGACGTCCTGTTCCAAGAAACATAGACAAGAGCCAACCCCAAGGTTACAATCTTCAAATTACAACTCGGACACCGAAGGCGCCAGATTTCAAATTTGAGCTTTTGCCGCTTCACTCGCCGCTACTAAGGCAATCCCTGTTGGTTTCTTTT

B12 - *Meyerozyma guilliermondii* MSRY_19

ID: KY952849.1

100% of identity

F

TAGCGGCGAGTGAAGCGGCAAAAGCTCAAATTTGAAATCTGGCGCCTTCGGTGTCCGAGTTGTAATTTGAAGATTGTAACCTTGGGGTTGGCTCTTGTCTATGTTTCTTGGAACAGGACGTCACAGAGGGTGAGAATCCCGTGCGATGAGATGCCCAATTCTATGTAAGGTGCTTTCGAAGAGTCGAGTTGTTTGGGAATGCAGCTCTAAGTGGGTGGTAAATTCCATCTAAAGCTAAATATTGGCGAGAGACCGATAGCGAACAAGTACAGTGATGGAAAGATGAAAAGAACTTTGAAAAGAGAGTGAAAAAGTACGTGAAATTGTTGAAAGGGAAGGGTTTGAGATCAGACTCGATATTTTGTGAGCCTTGCCTTCGTGGCGGGGTGACCCGCAGCTTATCGGGCCAGCATCGGTTTGGGCGGTAGGATAATGGCGTAGGAATGTGACTTTACTTCGGTGAAGTGTTATAGCCTGCGTTGATGCTGCCTGCCTAGACCGAGGACTGCGATTTTATCAAGGATGCTGGCATAATGATCCCAAACCG

R

AGTCCTCGGTCTAGGCAGGCAGCATCAACGCAGGCTATAACACTTCACCGAAGTAAAGTCACATTCCTACGCCATTATCCTACCGCCCAAACCGATGCTGGCCCGATAAGCTGCGGGTCACCCCGCCACGAAGGCAAGGCTCACAAAATATCGAGTCTGATCTCAAACCCTTCCCTTTCAACAATTTCACGTACTTTTTCACTCTCTTTTCAAAGTTCTTTTCATCTTTCCATCACTGTACTTGTTCGCTATCGGTCTCTCGCCAATATTTAGCTTTAGATGGAATTTACCACCCACTTAGAGCTGCATTCCCAAACAACTCGACTCTTCGAAAGCACCTTACATAGAATTGGGCATCTCATCGCACGGGATTCTCACCCTCTGTGACGTCCTGTTCCAAGAAACATAGACNAGAGCCAACCCCAAGGTTACAATCTTCAAATTACAACTCGGACACCGAANGCGCCAGATTTCAAATTTGAGCTTTTGCCGCTTCACTCGCCGCTAC

G12 - *Meyerozyma guilliermondii* N2-1

ID: MF148904.1

99% of identity

G12 - *Meyerozyma caribbica*

ID: KX507035.1

99% of identity

F

GTAGCGGCGAGTGANGCGGCAAAAGCTCAAATTTGAAATCTGGCGCCTTCGGTGTCCGAGTTGTAATTTGAAGATTGTAACCTTGGGGTTGGCTCTTGTCTATGTTTCTTGGAACAGGACGTCACAGAGGGTGAGAATCCCGTGCGATGAGATGCCCAATTCTATGTAAGGTGCTTTCGAAGAGTCGAGTTGTTTGGGAATGCAGCTCTAAGTGGGTGGTAAATTCCATCTAAAGCTAAATATTGGCGAGAGACCGATAGCGAACAAGTACAGTGATGGAAAGATGAAAAGAACTTTGAAAAGAGAGTGAAAAAGTACGTGAAATTGTTGAAAGGGAAGGGTTTGAGATCAGACTCGATATTTTGTGAGCCTTGCCTTCGTGGCGGGGTGACCCGCAGCTTATCGGGCCAGCATCGGTTTGGGCGGTAGGATAATGGCGTAGGAATGTGACTTTACTTCGGTGAAGTGTTATAGCCTGCGTTGATGCTGCCTGCCTAGACCGAGGACTGCGATTTTATCAAGGATGCTGGCATAATGATCCCAAACCGCCCGTCTTANNNNCCGG

R

CTTGATNNATCGCAGTCCTCGGTCTAGGCAGGCAGCATCAACGCAGGCTATAACACTTCACCGAAGTAAAGTCACATTCCTACGCCATTATCCTACCGCCCAAACCGATGCTGGCCCGATAAGCTGCGGGTCACCCCGCCACGAAGGCAAGGCTCACAAAATATCGAGTCTGATCTCAAACCCTTCCCTTTCAACAATTTCACGTACTTTTTCACTCTCTTTTCAAAGTTCTTTTCATCTTTCCATCACTGTACTTGTTCGCTATCGGTCTCTCGCCAATATTTAGCTTTAGATGGAATTTACCACCCACTTAGAGCTGCATTCCCAAACAACTCGACTCTTCGAAAGCACCTTACATAGAATTGGGCATCTCATCGCACGGGATTCTCACCCTCTGTGACGTCCTGTTCCAAGAAACATAGACAAGAGCCAACCCCAAGGTTACAATCTTCAAATTACAACTCGGACACCGAAGGCGCCAGATTTCAAATTTGAGCTTTTGCCGCTTCACTCGCCGCTACTAAGGCAATCCCTGTTGGTTTCTTTT

H5 - *Meyerozyma guilliermondii* DGC-G-z

ID: MG518185.1

100% of identity

F

CCTTAGTAGCGGCGAGTGAAGCGGCAAAAGCTCAAATTTGAAATCTGGCGCCTTCGGTGTCCGAGTTGTAATTTGAAGATTGTAACCTTGGGGTTGGCTCTTGTCTATGTTTCTTGGAACAGGACGTCACAGAGGGTGAGAATCCCGTGCGATGAGATGCCCAATTCTATGTAAGGTGCTTTCGAAGAGTCGAGTTGTTTGGGAATGCAGCTCTAAGTGGGTGGTAAATTCCATCTAAAGCTAAATATTGGCGAGAGACCGATAGCGAACAAGTACAGTGATGGAAAGATGAAAAGAACTTTGAAAAGAGAGTGAAAAAGTACGTGAAATTGTTGAAAGGGAAGGGTTTGAGATCAGACTCGATATTTTGTGAGCCTTGCCTTCGTGGCGGGGTGACCCGCAGCTTATCGGGCCAGCATCGGTTTGGGCGGTAGGATAATGGCGTAGGAATGTGACTTTACTTCGGTGAAGTGTTATAGCCTGCGTTGATGCTGCCTGCCTAGACCGAGGACTGCGATTTTATCAAGGATGCTGGCATAATGATCCCAAACCGCC

R

TCGCAGTCCTCGGTCTAGGCAGGCAGCATCAACGCAGGCTATAACACTTCACCGAAGTAAAGTCACATTCCTACGCCATTATCCTACCGCCCAAACCGATGCTGGCCCGATAAGCTGCGGGTCACCCCGCCACGAAGGCAAGGCTCACAAAATATCGAGTCTGATCTCAAACCCTTCCCTTTCAACAATTTCACGTACTTTTTCACTCTCTTTTCAAAGTTCTTTTCATCTTTCCATCACTGTACTTGTTCGCTATCGGTCTCTCGCCAATATTTAGCTTTAGATGGAATTTACCACCCACTTAGAGCTGCATTCCCAAACAACTCGACTCTTCGAAAGCACCTTACATAGAATTGGGCATCTCATCGCACGGGATTCTCACCCTCTGTGACGTCCTGTTCCAAGAAACATAGACAAGAGCCAACCCCAAGGTTACAATCTTCAAATTACAACTCGGACACCGAAGGCGCCAGATTTCAAATTTGAGCTTTTGCCGCTTCACTCGCCGCTACTAAGGCAATCCCTGTTGG

H9 - *Meyerozyma guilliermondii* 2A-1C315III

ID: MG736036.1

100% of identity

F

GCCTTAGTAGCGGCGAGTGAAGCGGCAAAAGCTCAAATTTGAAATCTGGCGCCTTCGGTGTCCGAGTTGTAATTTGAAGATTGTAACCTTGGGGTTGGCTCTTGTCTATGTTTCTTGGAACAGGACGTCACAGAGGGTGAGAATCCCGTGCGATGAGATGCCCAATTCTATGTAAGGTGCTTTCGAAGAGTCGAGTTGTTTGGGAATGCAGCTCTAAGTGGGTGGTAAATTCCATCTAAAGCTAAATATTGGCGAGAGACCGATAGCGAACAAGTACAGTGATGGAAAGATGAAAAGAACTTTGAAAAGAGAGTGAAAAAGTACGTGAAATTGTTGAAAGGGAAGGGTTTGAGATCAGACTCGATATTTTGTGAGCCTTGCCTTCGTGGCGGGGTGACCCGCAGCTTATCGGGCCAGCATCGGTTTGGGCGGTAGGATAATGGCGTAGGAATGTGACTTTACTTCGGTGAAGTGTTATAGCCTGCGTTGATGCTGCCTGCCTAGACCGAGGACTGCGATTTTATCAAGGATGCTGGCATAATGATCCCAAACCGC

R

GCAGTCCTCGGTCTAGGCAGGCAGCATCAACGCAGGCTATAACACTTCACCGAAGTAAAGTCACATTCCTACGCCATTATCCTACCGCCCAAACCGATGCTGGCCCGATAAGCTGCGGGTCACCCCGCCACGAAGGCAAGGCTCACAAAATATCGAGTCTGATCTCAAACCCTTCCCTTTCAACAATTTCACGTACTTTTTCACTCTCTTTTCAAAGTTCTTTTCATCTTTCCATCACTGTACTTGTTCGCTATCGGTCTCTCGCCAATATTTAGCTTTAGATGGAATTTACCACCCACTTAGAGCTGCATTCCCAAACAACTCGACTCTTCGAAAGCACCTTACATAGAATTGGGCATCTCATCGCACGGGATTCTCACCCTCTGTGACGTCCTGTTCCAAGAAACATAGACAAGAGCCAACCCCAAGGTTACAATCTTCAAATTACAACTCGGACACCGAAGGCGCCAGATTTCAAATTTGAGCTTTTGCCGCTTCACTCGCCGCTACTAAGGCAATCCCTGTTGG

H12 - Meyerozyma guilliermondii MSRY_19

ID: KY952849.1

100% of identity

F

TTAGTAGCGGCGAGTGAAGCGGCAAAAGCTCAAATTTGAAATCTGGCGCCTTCGGTGTCCGAGTTGTAATTTGAAGATTGTAACCTTGGGGTTGGCTCTTGTCTATGTTTCTTGGAACAGGACGTCACAGAGGGTGAGAATCCCGTGCGATGAGATGCCCAATTCTATGTAAGGTGCTTTCGAAGAGTCGAGTTGTTTGGGAATGCAGCTCTAAGTGGGTGGTAAATTCCATCTAAAGCTAAATATTGGCGAGAGACCGATAGCGAACAAGTACAGTGATGGAAAGATGAAAAGAACTTTGAAAAGAGAGTGAAAAAGTACGTGAAATTGTTGAAAGGGAAGGGTTTGAGATCAGACTCGATATTTTGTGAGCCTTGCCTTCGTGGCGGGGTGACCCGCAGCTTATCGGGCCAGCATCGGTTTGGGCGGTAGGATAATGGCGTAGGAATGTGACTTTACTTCGGTGAAGTGTTATAGCCTGCGTTGATGCTGCCTGCCTAGACCGAGGACTGCGATTTTATCAAGGATGCTGGCATAATGATCCCAAACCGCC

R

AGTCCTCGGTCTAGGCAGGCAGCATCAACGCAGGCTATAACACTTCACCGAAGTAAAGTCACATTCCTACGCCATTATCCTACCGCCCAAACCGATGCTGGCCCGATAAGCTGCGGGTCACCCCGCCACGAAGGCAAGGCTCACAAAATATCGAGTCTGATCTCAAACCCTTCCCTTTCAACAATTTCACGTACTTTTTCACTCTCTTTTCAAAGTTCTTTTCATCTTTCCATCACTGTACTTGTTCGCTATCGGTCTCTCGCCAATATTTAGCTTTAGATGGAATTTACCACCCACTTAGAGCTGCATTCCCAAACAACTCGACTCTTCGAAAGCACCTTACATAGAATTGGGCATCTCATCGCACGGGATTCTCACCCTCTGTGACGTCCTGTTCCAAGAAACATAGACNAGAGCCAACCCCAAGGTTACAATCTTCAAATTACAACTCGGACACCGAANGCGCCAGATTTCAAATTTGAGCTTTTGCCGCTTCACTCGCCGCTAC
